# Fundamental Biomaterial Considerations in the Development of a 3D Model Representative of Primary Open Angle Glaucoma

**DOI:** 10.3390/bioengineering8110147

**Published:** 2021-10-20

**Authors:** Hannah C. Lamont, Imran Masood, Liam M. Grover, Alicia J. El Haj, Lisa J. Hill

**Affiliations:** 1 School of Biomedical Sciences, Institute of Clinical Sciences, University of Birmingham, Edgbaston, Birmingham B15 2TT, UK; hcl954@student.bham.ac.uk (H.C.L.); imranmasood777@gmail.com (I.M.); 2 School of Chemical Engineering, Healthcare Technologies Institute, University of Birmingham, Edgbaston, Birmingham B15 2TT, UK; L.M.Grover@bham.ac.uk (L.M.G.); A.ElHaj@bham.ac.uk (A.J.E.H.)

**Keywords:** primary open angle glaucoma, trabecular meshwork, 3D in vitro models, Schlemm’s canal, mechanical properties, biomaterials

## Abstract

Glaucoma is a leading cause of irreversible blindness globally, with primary open angle glaucoma (POAG) being the most common subset. Raised intraocular pressure is an important risk factor for POAG and is caused by a reduction in aqueous humour (AqH) outflow due to dysfunctional cellular and matrix dynamics in the eye’s main drainage site, the trabecular meshwork (TM) and Schlemm’s canal (SC). The TM/SC are highly specialised tissues that regulate AqH outflow; however, their exact mechanisms of AqH outflow control are still not fully understood. Emulating physiologically relevant 3D TM/S in vitro models poses challenges to accurately mimic the complex biophysical and biochemical cues that take place in healthy and glaucomatous TM/SC in vivo. With development of such models still in its infancy, there is a clear need for more well-defined approaches that will accurately contrast the two central regions that become dysfunctional in POAG; the juxtacanalicular tissue (JCT) region of the TM and inner wall endothelia of the Schlemm’s canal (eSC). This review will discuss the unique biological and biomechanical characteristics that are thought to influence AqH outflow and POAG progression. Further consideration into fundamental biomaterial attributes for the formation of a biomimetic POAG/AqH outflow model will also be explored for future success in pre-clinical drug discovery and disease translation.

## 1. Introduction

**Figure 1:**
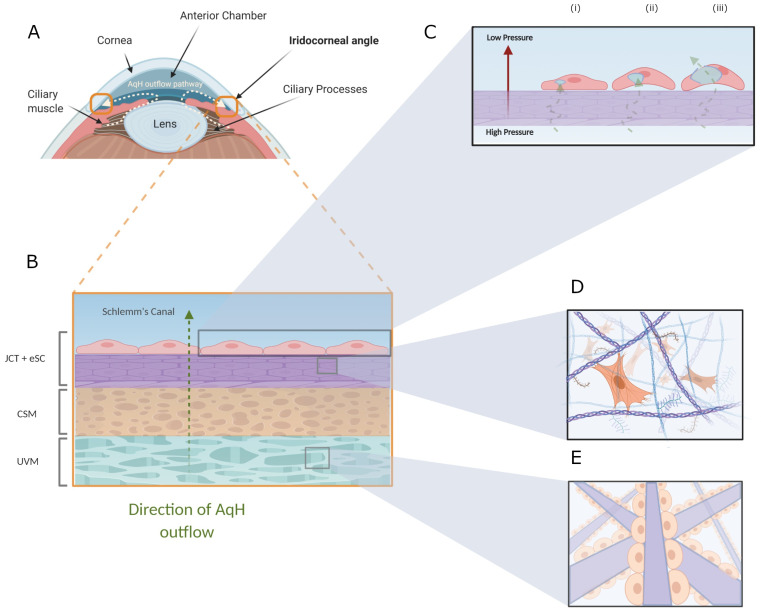
The AqH outflow pathway. (**A**) Diagram of the anterior chamber of the eye and direction of AqH movement towards the iridocorneal angle. The AqH outflow pathway within the iridocorneal angle is indicated by the bold orange square. (**B**) A magnified section of the AqH outflow pathway is presented with distinction and the 3 segments of the TM: Uveal Meshwork (UVM), Corneoscleral Meshwork (CSM), Juxtacanalicular Tissue and inner wall endothelia of the Schlemm’s canal (JCT and eSC). Green dotted arrow represents the direction of aqueous humour (AqH) flow through the TM and eSC, into the Schlemm’s canal. (**C**) eSC giant vacuole (GV) formation is presented: (i) Pressure builds on the basal side of the cell, opening micron-sized pores. (ii) AqH flows through the micron-sized pore, inducing a large cellular deformation (iii) Formation of GV structures, AqH enters into the lumen of the Schlemm’s canal. (**D**) Magnification of the JCT section, representing fibroblast-like TM cells sparsely embedded into a connective tissue matrix. (**E**) Magnification of the UVM section (also representative of the CSM section), with TM cells resting on collagen beams as a continuous monolayer, displaying an endothelial phenotype.

Glaucoma and its association with dysfunctional aqueous humour (AqH) flow through the anterior chamber of the eye has been observed in medicine for over a century [1,2,3,4]. This progressive neurodegenerative disease is recognised as the most common cause of irreversible blindness globally, with a projected increase from over 70 to 111.8 million affected by 2040, with the most prevalent form being primary open-angle glaucoma (POAG) [5,6,7]. Raised intraocular pressure is the major modifiable risk factor for POAG. Other factors include race (with increased risk in African-Caribbean), genetics, and vascular disorders [6,7]. The risk of developing POAG increases with the number of risk factors a patient possesses, with several genetic mutations having the potential to independently cause the disease [7]. However, due to POAG having a multifactorial aetiology, determining pathogenesis is complex. What is evident though, is that a sustained increase in intraocular pressure (IOP) is related to gradual visual loss as a consequence of the following events: [1] retinal ganglion cell death by apoptosis, [2] glaucomatous optic atrophy, and [3] progressive defect of visual field, inevitably leading to irreversible and complete vision loss [7,8]. This sustained increase in IOP results from abnormal AqH outflow resistance located primarily at the trabecular meshwork (TM), Schlemm’s canal (SC) (Figure 1), with further fluid impedance occurring within the collecting channels and aqueous veins [8]. While it is known that drainage of AqH through the TM/SC accounts for >80% of AqH outflow from the eye [9] and is a major regulator of IOP, there is still limited understanding of how the fundamental mechanisms of AqH outflow drive disease pathology.

In vivo animal and ex vivo models are currently the gold standard methods to investigate the pathobiology of POAG and are used to assess new therapies [10,11]. The most common in vivo models use mice, since they possess an ocular anatomy comparable to that of humans, and are cost effective [11,12]; however, critics have questioned the continued reliance on animal models, stating they are unreliable for faithfully mimicking the complexities of glaucoma pathogenesis, and hence are limited to helping successful translation of new therapies into the clinic [10,11]. It has been widely recognised within medical research and pharmaceutical testing that animal models have, overall, inherent limitations due to species variations in genetics and micro-architecture, subsequently falling short of recapitulating aspects of human disease and drug efficacy [13,14,15]. With an increasing need for more clinically translatable models of POAG, the interdisciplinary field of tissue engineering offers alternative approaches by applying the fundamental principles of cellular biology and biomaterial science to create functional, humanised 3D in vitro models [16,17]. Applying these principles will be key for the advancement of a 3D co-culture model that is representative of an AqH outflow system. Notably by permitting a more accurate investigation into dysfunctional fluid flow and disease manifestation, mimicking predominant risk factors such as IOP.

For POAG in vitro modelling, there have been significant advancements recently in regard to co-culture development, mimicking POAG-like scenarios, and drug responses [18,19,20]. Whereas the development of a 3D in vitro model which can reliably recreate the complex and reciprocal interactions between the TM and inner wall endothelia of the Schlemm’s canal (eSC) as a fluid flow model is still a great challenge within the field [21]. The TM and eSC are separate cell types that contribute to the development of POAG by simultaneously mediating AqH outflow [22,23,24,25,26]. Considering the limited knowledge of how this cellular crosstalk occurs, it is recognised that several biological factors and mechanical forces work in synchronicity for homeostatic AqH flow [27]. As the emergence of POAG pathogenesis cannot be attributed to a single tissue within the AqH outflow pathway and dysfunction arising from different sources, this highlights the need for an established co-culture model that is representative of this. The TM tissue is not a rigid structure, but a highly dynamic, avascular filtration system that has a multitude of roles, including filtering AqH of waste material [28], sensing and regulating IOP through mechanical stretch, and altering extracellular matrix (ECM) composition and deposition [29]. Alterations in the structure and composition of the TM ECM generates AqH outflow resistance, with IOP being a byproduct of this accumulation of resistance [30]. Current TM/eSC models focus on recapitulating specific aspects of natural tissue functionality or dysfunction, such as cell–ECM dynamics and uncontrolled ECM deposition. Although extensive research has been carried out, no single study exists which wholly mimics the physiologically relevant microenvironment and/or dynamic biophysical cues that are present in vivo (cyclic stress/strain, topography or elasticity) [18,31,32,33]. As contrasting types of biomaterials and fabrication techniques have been implemented for the formation of TM or eSC constructs, one major drawback is the inconsistency in the outputs measured. Subsequently, this lack of uniformity suggests the need for standardisation of in vitro model outputs for disease and in pre-clinical testing of POAG therapies. Doing so will not only enhance the efficiency in pre-clinical observations, but direct future POAG in vitro models towards translation into pharmaceutical industries.

In this review, we discuss the biological characteristics of the TM/eSC cells that are relevant for in the creation of a 3D in vitro model that has the potential to imitate AqH outflow and attributes of POAG. Furthermore, biomaterials used for the formation of current TM/eSC in vitro models and other relevant cellular studies will be reviewed, with specific aspects of these materials holding importance to mimic the appropriate cellular characteristics of the TM/eSC in vivo.

## 2. TM and eSC Biological Properties

**Figure 2:**
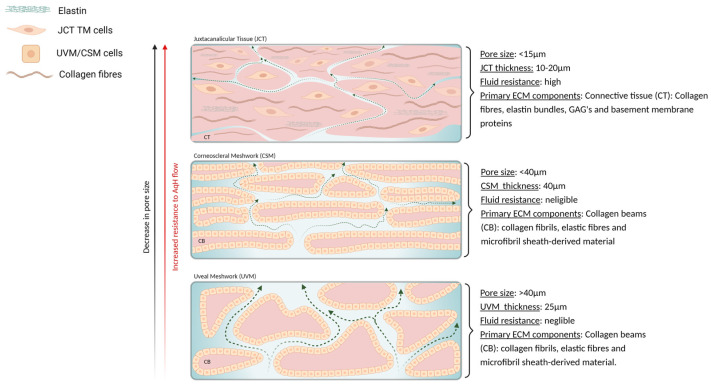
Schematic of TM structure and AqH flow. A differing structure is presented within each section of the TM tissue, with dotted arrows indicating direction of AqH outflow. UVM: The outermost section of the TM consists of randomly orientated collagen beams (CB) and larger pore size than other segments. CSM: Collagen beams become progressively thinner and more striated going deeper into the TM tissue; collagen beams run parallel to the Schlemm’s canal, and contain a markedly reduced pore size compared to the UVM. JCT: Deepest portion of the TM tissue has a contrasting structure to UVM and CSM, consisting of layers of connective tissue with cells embedded within the tissue, and comparatively small pore size (<20 µm).

Choice of scaffold design, material selection and fabrication techniques are critical considerations when developing an AqH outflow model, with several biological factors needing to be taken into consideration to encompass the distinct morphological and functional features associated with IOP regulation. The importance of ECM dynamics within the TM for optimal AqH fluid dynamics and healthy IOP regulation is stated frequently in recent research, as molecular organisation and relative of abundance of these ECM components, for example, glycosaminoglycan’s (GAG), vary throughout the TM segments and are thought to be primary influencers of AqH outflow dynamics [26,34]. Additionally, human and porcine tissue studies have observed heterogeneity in ECM components and microarchitecture throughout the TM when controlled by local TM cells that are stimulated by various mechanical cues [23,35,36,37,38,39]. With these factors altering IOP, it is dysfunctions in these homeostatic mechanisms that leads to disease manifestation. Morphologically, the TM consists of three distinctive layers (Figure 2), which differ in structure and function: the uveal meshwork (UVM), corneoscleral meshwork (CSM) and the juxtacanalicular tissue (JCT). Porosity and pore size is a significant factor in appropriate AqH outflow, with a gradient of pore size existing within the TM [24,40]; the outermost sections of the TM (UVM and CSM) are characterised by a high pore density and size ranging between 2 and 75 µm (Figure 2) [41,42], providing negligible outflow resistance [9,43,44]. The innermost part of the TM, the JCT, is widely regarded as a highly specialised area of connective tissue and not a meshwork, with a contrasting morphology, comparatively small pore size (<15 µm), and high resistance to fluid flow [8,45,46]. Studies in both human and primate TM tissue have noted this substantially contrasting structure and cellular characteristics, leading many scholars to hold the view that the JCT is the primary site of AqH outflow resistance and dysfunction during POAG pathogenesis [23,43,46,47]. With importance being placed on the JCT region, appreciation of the JCT ECM architecture, biochemical makeup and TM cellular characteristics will be further discussed for replicating AqH outflow systems and POAG in the following section.

The JCT displays a unique ECM organisation and composition, with local TM cells in the JCT exhibiting a distinctive fibroblast/myofibroblast behaviour that allows continuous ECM remodelling to occur. Evidence suggests that this constant ECM remodelling is akin to an unresolved ‘wound healing response’, altering the resistance by which AqH can exit the eye to compensate for fluctuations in IOP [23,35,48]. The JCT-TM cells are sparsely embedded between 2–5 layers of connective tissue, surrounded by an amorphous, collagenous 3D ECM that is filled with numerous basement membrane (BM) proteins and bioactive components (Figure 2) [23,28,34]. These components create a hydrated, negatively charged, BM-like ECM, composed of laminin, fibronectin, collagen type IV, proteoglycans and hyaluronic acid (HA) [23,49,50,51]. An important trait of TM cellular functionality, is their inherent plasticity to differentiate into different cell types, with the characteristics the cells display being highly dependent on the biochemical and biophysical cues they are exposed to, an aspect that will influence the development of a TM or JCT model [28,52,53,54,55]. It is thought that the JCT cells exhibit fibroblast/myofibroblast features (Figure 1D), which can transition into a predominantly endothelial/phagocytic phenotype within the UVM/CSM sections (Figure 1E) [28]. This presents an interesting perspective for future studies as to what particular biochemical or biophysical cues contribute to TM cellular behaviour. By understanding and fine-tuning of these effectors during scaffold development, this in turn will exploit wanted cellular attributes and allow for the stabilisation of the JCT cell phenotype and modification of an AqH outflow model. In vitro research that has contributed towards this notion in modulating cellular characteristics through material aspects will be discussed in greater detail within the following sections of this review.

Another important cell type in AqH outflow and POAG is the eSC, located adjacent to the JCT [24,25,56,57]; as the JCT generates resistance, the neighbouring endothelia also help to regulate resistance to outflow [58]. AqH outflow is a pressure-dependent system, as eSC cells uniquely sense pressure gradients from the basal-apical direction. When the AqH pressure on the basal side is high enough due to JCT resistance, it has been noted from both ex vivo and in vitro studies that a set series of events will occur: (1) the opening of transendothelial pores, facilitating AqH flow through the eSC cells, (2) the creation of large transcellular mechanical loads, and (3) the generation of large cellular protrusions known as ‘giant vacuole’ (GV) structures [59,60] (Figure 1C). It is recognised that the formation of these GV structures contributes to the tissue’s strikingly high hydraulic conductivity, and is a fundamental function necessary for healthy outflow [61]. Considering the dynamic behaviour in GV formation is a contributor to IOP homeostasis, with GV density and size being pressure dependent [59,60], future AqH outflow in vitro models must have the ability to replicate the 3D structural and biomechanical cues the eSC present, to account for the innate pressure variations seen in human eyes. Furthermore, a relevant 3D co-culture in vitro model that can express complex cellular communication and the relevant genetic attributes associated with AqH outflow regulation in vitro may develop resistance and regulate pressure gradient formation. As previously mentioned, the manner in which JCT-TM cells communicate with neighbouring eSC to generate resistance and wholly regulate outflow is still not clear. Nevertheless, the overall resistance measured within the AqH outflow pathway cannot be attributed to either tissue as single entities or as a whole when added in series [24]. It has been hypothesised that complex hydrodynamic interactions are believed to exist between the TM and eSC, creating the total pressure accumulation observed in previous research [24]. With a wealth of information to implicate eSC as a critical mediator of resistance and its dysfunction leading to POAG (due to changes in cellular behaviour) in both human ex vivo and in vitro studies [25,56,57,58,62]. The addition of eSC would be imperative for emulating a physiological relevant AqH outflow and POAG models. Thus, this review will further take into consideration the aspects of biomaterials that are advantageous for the incorporation of the eSC.

## 3. Current Biomaterial Approaches to Modelling the TM/eSC

The development of TM or eSC in vitro systems is still in its infancy, with only a limited number having been designed. As contrasting biomaterials and fabrication techniques have been applied for adapting TM/eSC in vitro models, this has led to new conceptual insights and demonstrated specific biomaterial properties that influence TM and eSC cell fate (Table 1). This section will further look into the physical and biochemical properties of biomaterials that hold significance in regulating cellular fate and hence, tissue functionality, for the formation of an AqH outflow/POAG co-culture in vitro model.

### 3.1. Biomaterial Composition

When forming in vitro cultures with ECM-remodelling cells, such as the JCT-TM cells, cellular interaction with the biomaterial and remodelling of the polymer network is necessary to allow for appropriate physiological analyses. With natural biopolymers having been largely used for the development of TM in vitro models and present advantageous properties for regulating connective tissue formation through constructive ECM remodelling [63,64,65,66], this section will focus only on studies that have applied natural polymers in the development of TM/SC models. 

Type I collagen is one of the most abundant fibrous proteins within the human body and also the backbone of the TM tissue [26], with a majority of in vitro TM models having applied type I collagen gels as a central scaffold [20,32,67,68,69]. As TM tissue contraction is thought to be a factor in facilitating AqH outflow and IOP regulation, type I collagen contraction assays are an established model for assessing TM cell response under various conditions [48,68,69,70]. JCT-TM cells possess smooth muscle-like characteristics [71], the extent of contractile properties that the TM cells express can be readily assessed by the change in the diameter of TM cell-laden type I collagen gels [72]. These key features have allowed the pathology of POAG to be mimicked through heightened type I collagen contraction in the presence of TM pro-fibrotic compounds; TGFβ-1 [68], fibronectin [20,48] endothelin-1 [69,70] and dexamethasone [20]. Moreover, visual relaxation of contracted type I collagen gels can be further detected through the application of known or potential POAG therapeutics: Rho kinase inhibitor and nitric oxide [20,68,73]. With the magnitude of collagen contraction suggested to be concentration and time dependent [20,48,68], dynamic and bidirectional responses can be observed in vitro and potentially beneficial for high-throughput testing of potential POAG therapeutics.

The effect that scaffold composition has on TM cellular characteristics has also been assessed and compared in different type I collagen–GAG composites [20,32,67] due to the importance of GAGs on tissue functionality and disease progression [36,37,74,75,76,77,78,79,80]. Initial observations proposed that the incorporation of chondroitin sulphate (CS) within a collagen scaffold would create an architecture similar to that in vivo for 3D culture models, owing to its presence within innate TM tissue [32,36]. The findings from Osmond (2017) established that type I collagen–GAG composites maintained TM cell metabolic activity for up to 2 weeks [32,67]. Osmond (2020) further illustrated a variation in TM cellular characteristics when cultured on scaffolds of differing collagen–CS or collagen–HA composites, with TM cellular outputs being assessed through fibronectin deposition and TM cellular metabolic activity [67]. The relevance of fibronectin as a marker is due to its crucial role in TM tissue functionality, influencing cellular survival and incorporation of other ECM proteins that are highly expressed in the TM, such as collagen type IV, laminin and fibrillin [81]. TM proliferation was deemed highest in collagen–CS and collagen-only scaffolds; in contrast, the genetic expression of fibronectin was more consistent in collagen–HA scaffolds over time regardless of microarchitecture [67]. A possible explanation for these conflicting outputs may be due to the GAG’s significantly different roles within TM tissue functionality and AqH outflow, as other studies have also highlighted HA as a promising GAG to incorporate into in vitro TM/eSC models [20]. HA being one of the most abundant GAGs within the TM and eSC, both cell types are rich in CD44 receptors that further allow dynamic cross-talk with the ECM [78,82,83], a loss of HA is also correlated with POAG progression [36,37,74,84]. The presence of HA can have multifaceted effects within the TM, such as immobilising large amounts of water and ions [85] for accumulative outflow resistance, regulating ECM protein synthesis, and mediating cell fate [36,37,74]. With CS being a sulphated GAG, it has been previously reported that CS can covalently attach to fibronectin at the cell surface to aid cell–ECM interactions [86], mediating cell survival [87] and consequently augmenting cellular metabolic activity and proliferation when compared to non-sulphated GAGs. Bearing this in mind, it must be noted that the presence of HA does not hinder a baseline of fibronectin or collagen type IV deposition in any of the collagen–GAG composites previously studied [20,67]. While it has been previously noted that HA can steadily regulate transcription of fibronectin [67], HA also has the ability to downregulate the assembly of such fibrillar components at the cell surface [66], suggesting that its presence can regulate fibronectin deposition into the local ECM environment, expressing antifibrotic effects [66]. In addition, eSC in vitro model studies have also reported beneficial effects of HA, contributing towards enhanced cellular proliferation and monolayer formation when chemically cross-linked with gelatin [88]. Overall, with the information presented, studies that suggested these differing cellular responses from CS– and HA–collagen scaffolds will inevitably cause a disparity in the cellular output data when assessing TM cultures in the short term (i.e., several days) [67]. However, considering the long-term impact of the type I collagen–GAG composites may hold more relevance if assessed for longer durations (i.e., several weeks) in the development of future POAG/AqH outflow models. With an increased presence of CS being associated with the pathology in POAG [36,37], it is proposed to be a central component in regulating AqH flow through the TM [39] and can readily facilitate ECM accumulation [86]. Ultimately, the occurrence of abnormal or unregulated fibronectin/GAG deposition has the potential to disproportionately alter ECM organisation, resulting in cellular dysfunction, aberrant ECM metabolism, and hydrodynamic abnormalities reflected in POAG pathology [34,89,90]. For this reason, caution must be taken with the application of certain bioactive components when creating healthy TM/eSC-like systems, as possible bias in cellular response will play a significant role in modelling JCT/AqH outflow models; furthermore, the addition of such components may cause expression of uncontrolled pathological POAG characteristics in vitro. Recently, the incorporation of elastin-like polypeptides into type I collagen–HA composites has also been developed to help overcome limitations of previous TM models for fully recapitulating the native TM tissue architecture and interactions [20]. It was stated that the incorporation of the particular ECM biopolymers similar to that found in the native JCT [23] would create a more biologically relevant TM model. This model was able to provide a more well-defined analyses of reciprocal TM-ECM interactions and drug testing for POAG. Although it was not assessed if presence of HA and elastin-like polypeptides was deemed favourable in enhancing TM cell characteristics compared to type I collagen scaffold alone, this particular study offered a more detailed investigation into 3D TM cell–ECM interactions and TM biomechanics under normal and glaucomatous conditions than previously researched in vitro models [20]

Other models have relied on Matrigel^®^ to form 3D TM cultures [33,53,91,92], being a gelatinous, basement-membrane (BM)-like matrix that is derived from Engelbreth-Holm-Swarm mouse sarcoma cells. While Matrigel^®^ has significant downfalls such as its tumorigenic origin and batch-to-batch mechanical variability [93], the BM-like matrix is similar to the ECM constituents that surround the JCT-TM cells and has been shown to provide stimulus for the restoration of TM cellular characteristics [23,33,53,94]. TM cells are highly sensitive to their biochemical environment and spatial position, with contrasting genetic expression of TM and associated stem cell markers being observed when cultured on Matrigel^®^ in 2D compared to 3D [53,91]. The exposure of TM cells to the BM- like matrix was observed to stabilise transcription of TM markers (AQP1, CHI3L1, MGP and AnkG) in comparison to other commonly used substrates used for the culturing of TM cells: fibronectin, gelatin, type IV collagen, and plastic [53]. Moreover, while 2D cultures were advantageous for only the expansion of TM cells, the restoration and expression of characteristic TM and stem cell markers were apparent when forming a 3D TM culture embedded within the Matrigel^®^. These findings have led other researchers to apply Matrigel^®^ for the formation of 3D TM cultures as drug screening technologies [33,91,92], modelling TM impairment in vitro by inducing oxidative stress. It was stated that enhanced restorative functions and gene expression within a 3D TM-Matrigel system was apparent in comparison to the 2D controls [91,92]. Ultimately, Matrigel^®^ alone may not be fully representative of the optimal conditions for mimicking JCT/AqH outflow features, as the studies only represent specific aspects of POAG by inducing mitochondrial dysfunction [33,91] and do not rule out the influence of other biophysical factors such as stiffness or topography. Despite this, the contrasting effects of 2D and 3D TM cultures has further reinforced the idea that the design of TM constructs would be more precise as an in vitro pre-clinical drug model in 3D form.

### 3.2. Topography

The effect of nanoscale to submicrometre topographic features on influencing TM cell alignment, migration and gene expression has been investigated in several studies [53,95,96,97]. Similar observations were noted: a loss of in vivo characteristic cell properties when cultured on tissue culture plastic or planar surfaces, such as myocillin [98], and TM/stem cell expression [53] and stabilisation of these necessary in vivo markers in the presence of randomised nanopatterns. This is of particular significance; as indicated previously, TM cells are encapsulated within their own BM-like matrix within the JCT, a matrix containing nanoscale features [99], and may be a key factor in regulating TM cellular fate in vivo. Thus, presenting similar cues in vitro will allow for the stabilisation of such in vivo-like traits and may be just as important for regulating the TM cells as the biochemical signals induced from the BM-matrix [53]. Synthetic polymers are popular for such studies due to the ease of developing refined physical features that mimic the physical attributes of the ECM [100], such as nano-grooved polyurethane surfaces containing varying ridge and groove nanopatterns [95] and non-aligned poly(etherurethane) urea electronspun nanofibers [96] being applied for TM cell culturing. It was illustrated that presence of these specific patterns had retained myocillin and verscian protein expression in comparison to TM cultures grown on tissue culture plastic or aligned surfaces [95,96]. This has led other studies to look deeper into the manipulation of TM cells through topographical features, with electrospun, non-aligned, nano-fibrous scaffolds containing poly L-lactic acid/polycaprolactone improving the differentiation of TM cells into glial and neural progenitor cells [97]. In addition to the embedding of TM cells into Matrigel^®^, a BM mixture that is rich in nanotopographical features [101] was deemed favourable for the expansion and manipulation of human TM cells into adipocytes, chondrocytes and endothelial cells [52,53].

Another important response to be considered in the design of an AqH outflow model in regard to topographical features is the manipulation of cellular morphology, as TM cells have the ability to express differing morphologies and differentiation states throughout the TM tissue, notably displaying rounded (endothelial), multipolar or spindle-shaped (fibroblast/smooth-muscle) [28,102]. Again, this may stem from the vastly differing topographical cues the localized TM cells are experiencing in vivo, with JCT-TM cells having a more elongated, spindle-shaped morphology [28]. Russell (2008) further correlated the presence of heightened in vivo markers to a significant change in TM cell elongation and actin filament alignment on anisotropically nanopatterned surfaces compared to planar surfaces [95]. Zhang (2018) continued to present similar findings of elongated, spindle-shaped TM cells when cultured on collagen type IV, fibronectin-coated surfaces, and 2D Matrigel^®^ cultures [53]. Another interesting observation to be noted from Zhang (2018) was the difference in TM cellular morphology from 2D to 3D culturing within Matrigel^®^ [53], with TM 3D cultures presenting a stellate morphology, echoing an upregulation of stem cells markers compared to 2D. Additionally, this study highlighted an important link between TM cellular phenotype and alterations in genetic expression. Research on the development of TM/POAG models has mostly focused on enhancing cellular proliferation or preserving TM cell viability, rather than replicating cellular morphologies and their relation to tissue functionality in vivo. An important consideration that needs to be addressed moving forward within the field is exactly what area of the TM tissue is being replicated, and whether the morphology/functionality of the TM cells represents appropriate dynamics to mirror AqH outflow or POAG formation. 

Other in vitro TM models have attempted to encompass aspects of topographical cues during scaffold development for creation of biomimetic TM/eSC systems [31,88]. Torrejon and colleagues used cytocompatible SU-8 epoxy photoresists to generate highly reproducible, microfabricated porous scaffolds, with significant efforts being made to match surface topography to TM tissue, ensuring that the model was as relevant as possible [31,103,104]. It was reported that the aligned porosity, pore size (12 µm) and beam width (7.3 ± 0.1 µm) of the SU-8 scaffolds produced matched the pore size (2–15 µm) and trabecular beam width found in vivo (5–12 µm) [31,45,99], providing the appropriate geometry for heightened TM cell proliferation, although the effects of this were negligible in eSC cultures, which have been demonstrated to grow easily on porous structures, regardless of dimensions [88]. There were some partial limitations to these studies: while said sub-micron features do produce robust TM cell cultures, the dimensions produced on the SU-8 scaffolds do not complement previous studies on maintaining important TM characteristic traits through non-aligned, nanostructured environments [53,95,96]. Furthermore, the TM cells were grown as a monoculture instead of a 3D culture, which could lead to deviations in proliferation rates, alterations in phenotypic traits and cellular senescence rates when compared to 3D cultures [105]. Whether or not topographical features in 2D can truly emulate the AqH outflow systems should be further considered, as this may significantly impact cellular responses during drug screening studies. Contrasting data produced by Osmond (2020) addressed the previous downfalls in topology for the generation of TM in vitro models, with the development of composite collagen–GAG scaffolds with differing structures and compositions to further understand the effect on TM proliferation and characteristics. While this model did not discuss the presence of nano-scale features, the results reinforced the general consensus that TM cells preferentially maintained stable proliferation and characteristics on non-aligned collagen scaffolds [67]. The current research discussed not only convincingly demonstrates the potent influence that random, nanoscale extracellular features have on TM cellular expression, but the inability of standard, 2D TM monolayer cultures to faithfully recapitulate these innate TM characteristics. Furthermore, the influence of topographical cues will be a significant contributory factor to the development of future in vitro TM models for faithfully modelling in vivo-like characteristics.

### 3.3. Porosity 

Emphasis has been placed on the heterogeneity of the TM tissue in terms of progressively diminishing pore size and increased ECM density from the outer to deeper layers of the tissue [8,40,41,106], with these attributes having been contemplated or attempted in several previous TM/eSC models [20,31,32,67,88]. Pore size and density is a major parameter in aiding AqH outflow for resistance generation [24,44,58,80] and potentially for maintaining TM cell viability [67]. When TM cells were cultured on top of scaffolds of an optimal pore size (12 µm), it was suggested to alter cellular characteristics, such as promoting the expression of in vivo markers (myocillin) and cellular viability [31]. Meanwhile, other TM models have estimated pore sizes within their hydrogel networks larger than those found within the in vivo JCT [20,67], with notably increased cell viability and dynamic cellular behaviour that is comparable to the JCT region [20]. Likewise, enhancing pore alignment within type I collagen/GAG scaffolds [67] has also been shown to positively effect TM cellular proliferation over 28 days compared to smaller pores, likely due to efficient nutrient/oxygen perfusion and higher surface area for cellular attachment. Conversely, TM cells cultured within collagen–GAG constructs that lacked pore alignment were preferable in maintaining and enhancing long-term TM cellular proliferation and viability. 

The discrepancy in pore size or porosity having a negligible effect on influencing TM cellular characteristics may support the assumption that these features are not dominating factors in influencing JCT formation in vitro. Nonetheless, the following parameters will be critical in the development of a physiologically relevant in vitro fluid flow, AqH outflow system by regulating resistance generation through the 3D cell culture constructs. It is believed that AqH outflow resistance results in pressure gradient formation at the interface of the JCT and eSC, with the porous characteristics of these tissues affecting the magnitude of resistance accumulated [24,46,90]. Current theoretical models of TM have demonstrated the effects of JCT and eSC porosity on AqH outflow by applying Darcy’s model (flow of fluid through a porous medium) [58,80], establishing the dependence of TM porosity and permeability on regulating IOP [107]. Therefore, adaptation of pore size and density will be paramount for developing an appropriate hydrodynamic relationship between the JCT and eSC to mimic the appropriate AqH outflow cues in vitro. As previously stated, Torrejon (2013) developed in vitro outflow TM/eSC models with established pore sizes (12 µm); this was said to be beneficial for the development of in vivo-like cellular monolayers; moreover, the studies were also able of recording differences in fluid flow when inducing a pathological state and introducing therapies [31,104]. A current gap within the field is that few studies have introduced 3D TM cell culture constructs into fluid flow systems. Another area of future research that would be beneficial is the formation of a 3D TM construct containing small, diverse pore architecture and that can be incorporated into a closed, fluid flow system [31,104]. This may demonstrate more dynamic mechanical cues than current TM/eSC outflow models, including the formation of localised pressure build-up within the 3D TM construct and subsequent pressure gradient generation that is independent of external factors. This has been reflected recently, through the application of a self-assembling peptide hydrogel system, MAX8B, suggesting enhancing the formation of a 3D biomimetic TM model [18]. With the incorporation of RGD peptide motifs, TM cellular attachment is set through integrin-mediated binding [18]. Nonetheless, while synthetic hydrogels lack attributes suitable for the development of an AqH model, the study induced fluid flow through the 3D porous TM-cell-laden scaffold, detecting variable and dynamic pressure gradients throughout the scaffold in both healthy and glaucomatous conditions [18]. This study may have shown promising results in paving the way for the imitation of fluid dynamic implications as found in TM tissue during POAG development and potentially homeostatic mechanisms through mechanoregulation by TM cells when sensing pressure.

### 3.4. Mechanical Properties

Several studies have indicated that there may be a significant correlation between TM tissue stiffness and AqH outflow facility, playing an important role in IOP stability [89,108,109]. An initial study by Last et al. (2011) found the local compressive modulus of human TM tissue to be 20 times stiffer in glaucomatous donors (80 ± 32.5 kPa) compared to healthy donors (4.0 ± 2.0 kPa) using atomic force microscopy (AFM) [89]. The application of AFM has been further favoured in determining the Young’s modulus of TM tissue in animal studies [108,109] to determine a significant relationship between TM mechanical properties and AqH outflow facility. In contrast, bulk tensile stiffness of human TM has been measured under uniaxial tension and proposed to have an average Young’s modulus (515 ± 136 kPa) [110] four times higher than glaucomatous human TM (125 ± 14 kPa) [111]. While it is evident that the discrepancy in moduli within these studies can be associated with the use of different measuring techniques [112], and the testing of opposing parts within the TM JCT [89], CSM [110,111], it could also be considered that the TM possesses anisotropic properties due to the collagen fibre orientation present, leading to a difference in compliance when applying opposing techniques for assessing TM mechanical properties, and possibly giving more insight into TM mechanical functionality [113,114]. This is particularly relevant, as the extent of collagen orientation effects the load-bearing properties of tissues, with the level of supercoiled collagen structure formed being key to this load-bearing capacity [115,116]. Abnormal collagen fibrillogenesis has been depicted in mice that are deficient in the same GAG’s as POAG patients [117,118], causing altered mechanical properties and similar markedly reduced tensile strength compared to glaucomatous TM tissue [118]. To add weight to this argument, animal models with known mutations in type I collagen to mimic POAG presented atypical collagen fibre formation, causing subsequent increased AqH outflow resistance and IOP [119,120]. All things considered, particular aspects of TM tissue components and formation of those components that alter the mechanical properties of TM tissue may be more relevant towards to the formation of an AqH outflow system or POAG model than the bulk/local mechanical properties themselves. 

TM in vitro model studies have referenced initial AFM data from human samples [89] as a relevant comparison for the substrate elasticity of TM biomimetic constructs during bulk compression or frequency sweep–strain testing [18]; however, there are several limitations to these findings. Firstly, post mortem eyes ranging from 4 to 30 days post death were tested, leading to considerable variability within data due to likely protein degradation over time [89]. In addition, the testing of excised tissue does not truly represent stiffness in vivo, as the TM tissue is under tension from the ciliary muscle attachments [106,121], the tension exerted from the muscle to the TM tissue will alter the true value of TM stiffness compared to post-mortem tissue [122]. Due to these downfalls, obtaining in vivo TM elastic modulus when inside the eye has also been attempted using optical coherence tomography (OCT), with a suggested average value of 128 kPa in healthy tissue [122], around 30 times stiffer compared to the previous AFM studies performed [89]. Thus, defining and relating the stiffness of TM tissue studies to future TM/eSC models should be deliberated upon cautiously in regard to optimising the hydrogel stiffness or mechanical loading for the formation of an AqH outflow model. It is important to recognise that it will also have significance by further permitting measures of cellular mechanotransduction and expression of particular phenotypic characteristics, as stabilisation of specific cellular characteristics will be imperative for the formation of an accurate AqH outflow model.

The notion of stiffness or biomechanical cues affecting eSC or TM cellular functionality has been meticulously investigated and validated [29,56], as cells in vivo are consistently subjected to oscillating mechanical loads naturally within the eye due to changes in IOP. Increasing the magnitude of external forces exerted upon the cells led to genotypic and phenotypic changes that are known to contribute to the pathogenesis of POAG [25,123,124]. Type I collagen coated polyacrylamide (PA) gels of varying stiffness (1–75 kPa) have been largely used in both TM and eSC culturing [25,125,126], illustrating the impact of substratum stiffness on TM/eSC functionality, modulating several characteristics such as actin stress fibre formation, focal adhesion size, cytoskeletal contractility and expression of several genes implicated in POAG [25,55,125,126]. When TM or eSC cells were subjected to static or cyclic biaxial strain when cultured on type I collagen [127,128,129,130,131,132,133] or laminin coated [134] PDMS, it was observed that the rate or magnitude of strain at differing time intervals significantly altered genes involved in cellular stress and ECM remodelling [127,128,131]. As TM cells experience pulsatile mechanical stress, stretch–strain and static models are intended to mimic these changes; however, they may not be wholly accurate [29,135]. The current eSC and TM models looking into these representative biomechanical cues are simplistic approximations of the dynamic, physiological conditions the cells experience, which is likely mediated by multiple signalling systems and interconnected pathways [27,129]. Ideally, future AqH outflow co-culture models should be exposed to dynamic biophysical cues within a 3D environment and subjected to more than one physical force for reliable data. Lastly, something to be highlighted for future studies is the contrasting differences between the culturing methods that have been performed during comparative assessments between 2D and 3D TM cell cultures; 2D cultures are grown on plastic or glass, in comparison to the TM cells, which are embedded into soft, viscoelastic scaffolds [20,91]. For example, as the local elastic modulus of Matrigel^®^ is 450 Pa [136] and traditional polystyrene tissue culture plastic possesses an elastic modulus >1 GPa [126], and there is a vast amount of information supporting the sensitivity of TM and eSC cells to differing elastic moduli of materials, this would be likely to alter gene and protein expression [55,126]. Standardization of similar culturing environments and mechanical characteristics would be favourable for creating more defined parameters for the assessment of cellular output in regard to the spatial positioning of TM cells.

**Table 1:** Biomaterial applications for in vitro TM and eSC models.

Materials	Material Classification	Cells	In Vitro Model	Outputs Measured	Pros (P) and Cons (C)	Author(s)
MAX8B	Shear thinning peptide hydrogel	Human TM cells	3D TM model drug testing platform	Cytoskeletal and ECM protein expression, cell viability, biomaterial stiffness and permeability	P: Fluid flow system, tuneable material properties, 3D culture, dynamic response C: Lack of ECM remodelling capacity	[18]
Type I Collagen + Hyaluronan + elastin like polypeptides	Protein/GAG/peptide--based hydrogel composite	Human TM cells	3D TM/glaucomatous model drug testing platform	Cell proliferation, viability, hydrogel contraction analysis, scaffold microstructure, actin cytoskeleton formation, gene expression, fibronectin protein expression, elastic moduli	P: ECM remodelling capacity, dynamic response, tuneable material properties, 3D cell culture C: static fluid system	[20]
Poly acrylamide (PA) (Type I collagen coated)	Synthetic polymer	Human TM cells /human eSC	Matrix stiffness	Genetic expression, actin stress fibre formation, cell spreading, focal adhesion size, cellular contractility, subcortical stiffness, cellular biomechanics	P: Controlled cellular characteristics, controlled material properties C: 2D culture, lack of ECM remodelling capacity	[25,55,125,126]
SU-8 epoxy photoresist + 1% Gelatin	Epoxy-based polymer (negative photoresist) + protein -based hydrogel	Human TM cells/human eSC	TM outflow system/ glaucomatous TM outflow system	Cell viability, TM marker expression, ECM protein expression, actin cytoskeleton formation, phagocytosis assay, gene expression, outflow facility, material pore size	P: fluid flow system, topographical cues, co-culture C: Fixed material parameters, 2D culture	[31,103,104]
Type I collagen + chondroitin sulphate	Protein/GAG--based hydrogel composite	Porcine TM cells	TM model	Elastic moduli, material pore size, GAG quantification, gene expression, cell viability and proliferation, fibronectin gene expression, fibronectin protein expression	P: tuneable material properties, ECM remodelling capacity C: static fluid system, decreased biomaterial retention, 2D culture	[32]
Matrigel^®^	Basement membrane-derived extract	Human TM cells	3D TM model drug testing platform/Gene manipulation	Gene expression; TM markers, stem cell markers, inflammatory cytokine markers, apoptotic markers. Actin cytoskeleton formation, cell viability, reactive oxygen species production	P: 3D culture, topographical cues, controlled cellular characteristics, fluid flow system C: Lack of ECM remodelling capacity, dynamic response and tuneable materials properties	[33,53,91,92]
Type I collagen + HA/HA+CS	Protein/GAG--based hydrogel composite	Human TM cells	TM model	Scaffold architecture, material pore size, GAG quantification, cell viability and proliferation, fibronectin gene expression, fibronectin protein expression	P: ECM remodelling capacity, tuneable material properties, topographical cues C: static fluid system, 2D culture, decreased biomaterial retention	[67]
Type I Collagen	Protein-based hydrogel	Human TM cells	Collagen contraction assays	Collagen contraction analysis, gene expression, protein expression, actin cytoskeleton formation, cell motility	P: ECM remodelling capacity, dynamic response, tuneable biomaterial properties C: static fluid system, 2D culture	[67,68,69,70,72,73]
SU-8 epoxy photoresist + Extracel™	Epoxy-based polymer (negative photoresist) + protein/GAG -based hydrogel (Thiol-modified HA and gelatin)	Human eSC	eSC outflow system	Cell viability, cellular characteristics, genetic expression, ECM protein expression, material pore size	P: fluid flow system, controlled cellular characteristics C: Fixed material parameters	[88]
Poly (etherurethane)	polymeric elastomer	Human TM cells	Topographical cues/Gene manipulation	Expression of characteristic TM protein expression	P: tuneable material properties, topographical cues, controlled cellular characteristics C: 2D cultures, Lack of ECM remodelling capacity, dynamic response	[95,96]
Poly L-lactic + Polycaprolactone	Thermoplastic polyester + thermoplastic polyester	Human TM cells	Gene manipulation	Gene expression, cellular protein expression, scaffold microstructure, fibre diameter,	P: tuneable material properties, topographical cues, controlled cellular characteristics C: Lack of ECM remodelling capacity, dynamic response	[97]
Polydimethylsiloxane (PDMS) (Type I Collagen or Laminin)	Silicone-based organic polymer/elastomer	Human/bovine TM cells	Dynamic or static cyclic stress/strain	Genetic expression of cellular stress proteins, ECM proteins and ECM remodelling proteins	P:Controlled material properties C: lack ofECM remodelling capacity, 2D culture	[127,128,129,130,131,132,133,134,135]

## 4. Future Perspectives and Potential Limitations

While the current TM/eSC in vitro models discussed have particular strengths and provide novel insights into biomaterial applications for the manipulation and formation of TM/eSC co-culture models, what is evident is the need for a 3D cell co-culture model that recreates dysfunctional AqH outflow, emulating the primary risk factor associated with POAG pathology. But doing so will be no easy feat, as several biological and biophysical factors must be applied to imitate the intricacy of TM/eSC tissue functionality and cellular cross-talk for regulation of pressure-induced fluid outflow. One promising avenue for the development of such a device is through organ-on-a-chip (OOAC) technologies [137]. OOAC technologies are defined as microfluidic, biomimetic systems that can recapitulate the environment of a physiological organ through several key parameters that hold significance for the development of an AqH outflow system: shear stress [138], cell patterning [139], cyclic stress/strain [140], and pressure gradient formation [141]. Such a device would potentially allow the application of several influential cues to be induced simultaneously, placing both TM and eSC in a dynamic state to regulate fluid outflow. Furthermore, OOAC applications are favoured in the production of high-throughput pre-clinical drug testing studies, further allowing the translation of POAG in vitro models into industry.

Nevertheless, such a strategy does not escape some major drawbacks that are still prevalent within the field of POAG in vitro models, and which will remain as limitations for an OOAC device. Firstly, one notable issue with the development of an OOAC POAG system, mirroring the complexity of the non-uniform, segmental outflow that occurs within the anterior chamber of the eye [142] and its likely effects on ophthalmic drug distribution [143]. Recapitulating this will present difficulties in terms of developing such complex hydrodynamics within an OOAC system. AqH flow through the TM tissue, having been previously discussed, may be attempted through the manipulation of porosity within the 3D construct, gaining the capacity to generate dynamic pressure gradients equivalent to the in vivo JCT and activating the eSC within the co-culture to induce fluid outflow. Another major drawback is that most in vitro models can only induce glaucomatous effects by introducing pro-fibrotic compounds such as TGFβ-1, TGFβ-2 or dexamethasone [20,68,144]. This would continue to be the case for current OOAC applications due to harnessing glaucomatous cells from patients having proven difficult, with no 3D in vitro model containing diseased, patient-derived cells currently published [145]. POAG is a disease that manifests due to genetic and environmental factors [146], characteristics that cannot be matched with the induction of pro-fibrotic compounds to create POAG-like scenarios. This could give rise to a lack in genetic diversity or impairment within drug metabolism that can occur from POAG patient cells [147]. These factors will inevitably impact the efficiency and effectiveness of PAOG models, as genetic diversity within any humanised in vitro system will be advantageous for the screening of toxic activities and variations in drug metabolism [148]. On the whole, while these are known challenges within the field, it should also be emphasised that these current limitations are also possible opportunities for future research. By tackling the issue of creating more physiologically relevant 3D in vitro models with multiple biophysical cues and the propagation of patient glaucomatous cells, space would be created for research towards patient-specific models.

## 5. Conclusions

Overall, the research performed to date on the development on TM/eSC/POAG models has focused on emulating more refined features of disease pathology rather than encompassing several known biological and biophysical factors simultaneously that contribute to disease manifestation to impede AqH outflow from the eye. The factors include: JCT cell–ECM dynamics, TM cellular phenotype, TM/eSC crosstalk, optimal porosity/permeability, stiffness, cyclic stress/strain and fluid flow within a 3D system. For this to occur, several design considerations and biomaterial aspects must be considered when developing an in vitro model that can imitate AqH outflow and POAG; aspects such as geometric control of the biomaterials for manipulation of cellular phenotype and genetic expression. Dynamic porosity for the generation of convoluted fluid flow and resistance generation to induce pressure gradients across the eSC. Furthermore, fine-tuning of the biomechanical cues and material composition needs further research and contemplation, with both having major effects on protein expression and interaction with the extracellular environment, this will likely alter the outcome of any in vitro model. Finally, it may be possible for all factors and design considerations to be applied synchronously to form a dynamic, 3D, fluid flow in vitro system through the application of OOAC devices. This will open space within the field of research for enhanced understanding into POAG manifestation and AqH outflow physiology. Further providing the opportunity for such novel pre-clinical 3D in vitro models with high-throughput status and standardised outputs, such as changes in fluid flow and pressure regulation, to be translated more efficiently from academia into the pharmaceutical industries.
